# The Ex-Press Mini Glaucoma Shunt: Technique and Experience

**DOI:** 10.4103/0974-9233.56226

**Published:** 2009

**Authors:** Steven R. Sarkisian

**Affiliations:** From the University of Oklahoma, Dean McGee Eye Institute, Glaucoma Service, 608 Stanton L. Young Blvd, Oklahoma City, Oklahoma 73104

**Keywords:** Glaucoma, Implantation, Surgical Technique

## Abstract

The Ex-Press Mini Glaucoma Shunt has been available internationally for almost a decade with almost 35,000 implantations world wide. The device shunts aqueous from the anterior chamber to a subconjunctival reservoir in a similar fashion as trabeculectomy, without removal of any sclera or iris tissue. Developments in ophthalmic surgery have been focused on smaller incisions to improve patient outcomes and visual recovery. The Ex-Press is an example of these developments. This article will review the surgical technique for implanting the Ex-Press Mini Glaucoma Shunt and will highlight the clinical experience with the device.

## BACKGROUND

**Figure 1:**
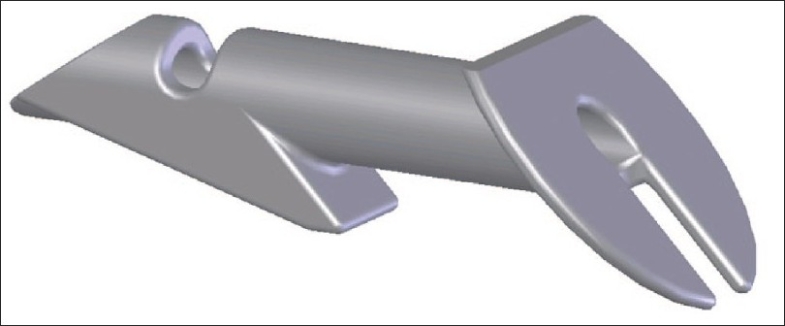
The Ex-Press Mini Glaucoma Shunt. Model P-50

The Ex-Press Mini Glaucoma Shunt [Figure 1] was originally developed by Optonol, Ltd. (Neve Ilan, Israel) for implantation under the conjunctiva for controlling intraocular pressure (IOP). This biocompatible device is almost 3 mm long with an external diameter of approximately 400 microns.^1^ It is a non-valved, MRI compatible, stainless steel device with a 50 micron lumen. It has an external disc at one end and a spur-like extension on the other to prevent extrusion.

The original unguarded technique of implantation under the conjunctiva caused numerous complications including hypotony, erosion, and extrusion of the implant. These complications have been well described in the literature.^2^–^12^ Endophthalmitis has also been associated with an exposed implant.^7^

To avoid complications associated with placement of the device under the conjunctiva, Dahan and Carmichael proposed implanting the device under a scleral flap.^13^ This technique has almost completely eliminated the erosion complication and has been demonstrated to have a lower rate of hypotony than trabeculectomy.^14^ Since 2003, Optonol, Ltd. has recommended all users of the device to only implant the device guarded under a scleral flap.

## SURGICAL TECHNIQUE

Topical or retrobulbar anesthesia is administered depending on patient selection and surgeon preference.

### Step 1

A standard fornix or limbal-based conjunctival incision is performed to gain exposure to the scleral bed adjacent to the limbus. Gentle cautery is performed in this area.

### Step 2

A scleral flap is created in a similar manner performed with a standard trabeculectomy. Care is taken to dissect the flap up to clear cornea. Anti-fibrotic agents can be applied either before or after the creation of the scleral flap in the usual manner based on the surgeon's preference.

### Step 3

A temporal paracentesis is created through the cornea.

**Figure 2:**
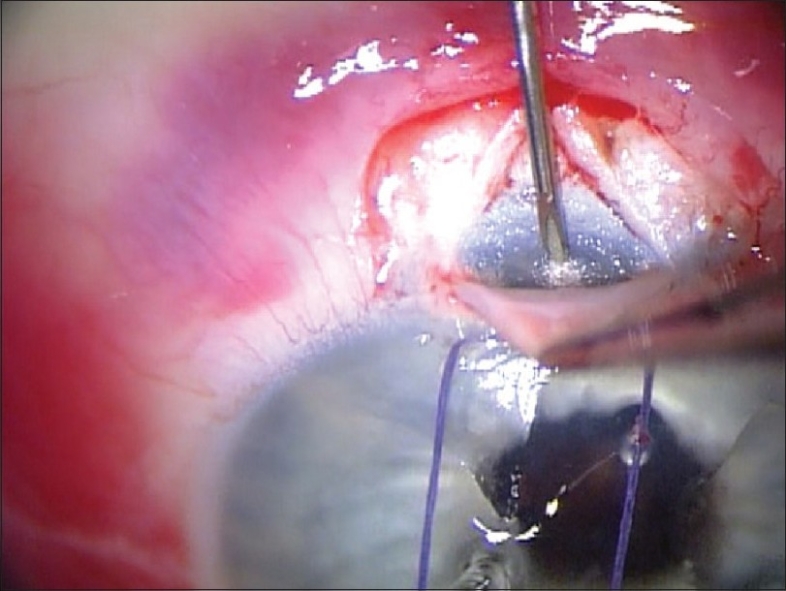
Creation of the wound for the Ex-Press Mini Glaucoma Shunt using a 26 Gauge Needle

The scleral flap is lifted and care is taken to identify the center of the “blue line” adjacent to clear cornea which corresponds to the location of the trabecular meshwork. A 26 gauge needle is inserted into the anterior chamber through the center of the “blue line” at an angle parallel to the iris plane. [Figure 2] The needle is removed. There must not be any lateral movement of the needle as this will cause aqueous to flow around the implant.

### Step 4

**Figure 3:**
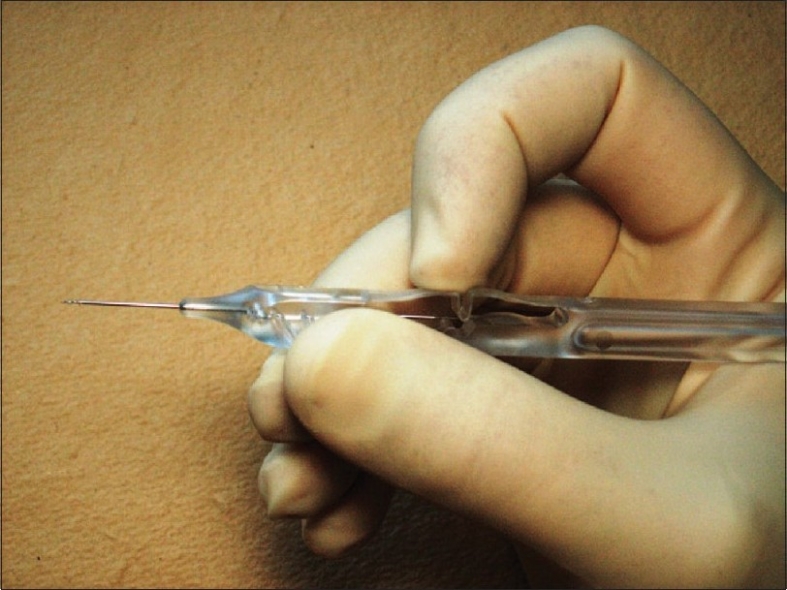
The Injection System for the Ex-Press Mini Glaucoma Shunt

The Ex-Press shunt is preloaded on an injector.^15^ Fitted into the lumen of the shunt is a metal rod that is attached to the end of the injector [Figure 3].

**Figure 4:**
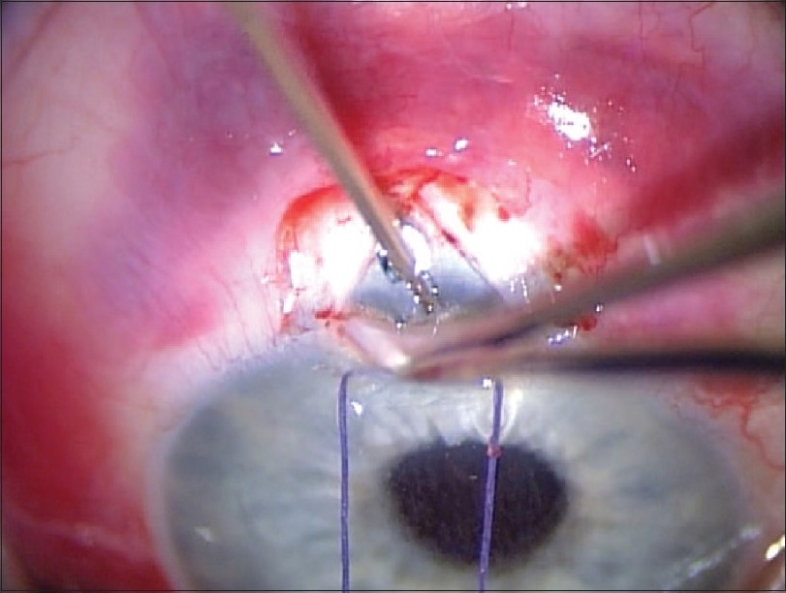
Insertion of the Ex-Press Mini Glaucoma Shunt

The shunt is then placed in the anterior chamber through the ostium created with the needle. The angle of entry with the shunt is the same as the angle used to make the ostium [Figure 4]. The shunt is inserted all the way into the wound making the plate flush with the scleral bed. In a similar fashion as a standard punctual plug inserter, the injector has an area on the shaft that is then depressed which retracts the metal rod in the lumen of the shunt. This allows the injector to be free from the lumen of the shunt.

### Step 5

The scleral flap is then sutured in place using a 10-0 nylon suture with a spatulated needle. One to three sutures are typically required depending on the flow which can be tested by inflating the anterior chamber with balanced salt solution with a 27 or 30 gauge canula through the temporal paracentesis.

### Step 6

The conjunctiva is then meticulously closed with the surgeon's suture of choice. I typically use a 9-0 polyglactin suture on a BV or VAS needle in a running or interrupted fashion. A fluorescein strip is used to make certain the wound is water tight.

## CLINICAL EXPERIENCE

**Figure 5:**
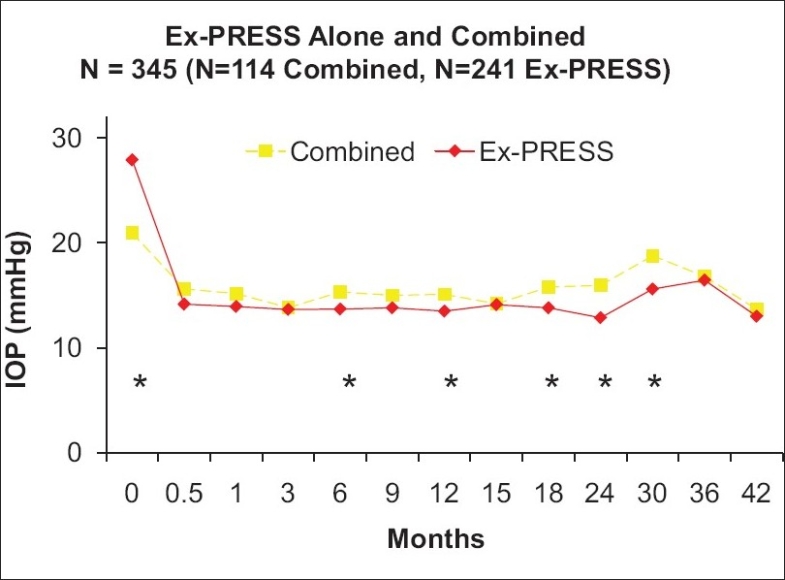
IOP measurements over time. Adapted from Kanner, *et al*. Ex-Press Miniature Glaucoma Device Implanted Under a Scleral Flap Alone or in Combination with Phacoemulsification Cataract Surgery

Thus far, the largest published case series of the Ex-Press Mini Glaucoma Shunt was by Kanner *et al.*^16^ This paper was a comparative consecutive case series of 345 patients, 231 eyes treated with Ex-Press implant under scleral flap alone and 114 eyes treated with Ex-Press implant under scleral flap combined with phacoemulsification. At 3 years after surgery, surgical success was 94.8% and 95.6% in the Ex-Press and combined groups, respectively. Compared with baseline values, the postoperative intraocular pressure [Figure 5] and number of glaucoma medications were significantly lowered in both groups. The change from baseline IOP was significantly greater after Ex-Press implant alone compared with combined surgery. Of interest, the most common device-related complication was obstruction of the tube (1.7%), which was treated successfully with Nd:YAG laser. It was not obstruction with iris tissue, as one might expect, but rather with fibrin in patients with a history of inflammation.^16^

It has been demonstrated by a previous paper by Maris *et al*. that there is a lower incidence of hypotony with the Ex-Press compared with trabeculectomy.^14^ Maris *et al*. demonstrated a 32% hypotony rate in the trabeculectomy group and 4% in the Ex-Press group.^14^ In Kanner *et al*., there was 15.6% with hypotony (IOP <5mm Hg) in the first week in the Ex-Press alone group, and 7.9% with hypotony in the first week in the combined group. All of these instances of hypotony during the early postoperative period resolved spontaneously. None of the eyes developed flat anterior chamber with lens-cornea touch.^16^

It is postulated that the lower rate of hypotony is from the resistance to flow that is offered by the 50 micron lumen of the shunt. Whereas, with trabeculectomy, the smallest scleral punch that is manufactured is approximately 750 microns and that is only if one punch alone is used to make the incision. It is clear that scleral sutures offer much of the resistance to flow with either trabeculectomy or the Express shunt; however, with the latter it is likely that both the small lumen of the implant and the suture tensioning offer resistance.

## THE TRANSITION TO SMALL INCISION SURGERY

Ophthalmic surgery has evolved over the last several decades into sophisticated microsurgery involving continually smaller incisions. This has been well demonstrated by developments in cataract surgery with phacoemulsification through small self-sealing clear cornea incisions and foldable intraocular lenses inserted using injectors. This evolution has also been shown in retina surgery with the advent of 25 gauge vitrectomy. It therefore follows that a similar evolution take place in glaucoma surgery with smaller, more precise wounds with more predictable and reliable results.

The Ex-Press shunt is on the forefront of this evolution toward smaller incision glaucoma filtration surgery. Although, there is an added cost to using the Ex-Press rather than trabeculectomy, I believe there are patient safety issues that are provided by the Ex-Press that make the added cost worthwhile. Moreover, it can be argued that fewer return visits to the operating room will occur with the lower rate of hypotony afforded by the Ex-Press. Examples of these benefits include fewer revisions for over-filtering blebs and much less frequent draining of choroidal effusions.

For the last decade there has been a philosophic paradigm shift in the surgical treatment for glaucoma with a desire to restore the natural outflow of aqueous humor via Schlemm's canal; however, none of these newer surgeries have been demonstrated to lower IOP as much or as reliably as guarded filtration surgery. Although not a paradigm shift regarding location of outflow, the Ex-Press offers a modification of outflow that is consistent with the general shift in surgery toward smaller incision microsurgery, affording easier utilization of topical anesthesia, less inflammation, greater predictability with less hypotony, and more rapid visual recovery.
